# Correction: Kebe et al. A Low-Phase-Noise 8 GHz Linear-Band Sub-Millimeter-Wave Phase-Locked Loop in 22 nm FD-SOI CMOS. *Micromachines* 2023, *14*, 1010

**DOI:** 10.3390/mi16020211

**Published:** 2025-02-13

**Authors:** Mamady Kebe, Mihai Sanduleanu

**Affiliations:** 1 School of Electrical Engineering and Computer Science (EECS), University of Ottawa, Ottawa, ON K1N 6N5, Canada; 2 System on Chip Center, Khalifa University of Science and Technology, Abu Dhabi P.O. Box 127788, United Arab Emirates; mihai.sanduleanu@ku.ac.ae

## Error in Affiliation

In the published publication [1], Dr. Mihai Sanduleanu’s affiliation is corrected to “System on Chip Center, Khalifa University of Science and Technology, Abu Dhabi P.O. Box 127788, United Arab Emirates”.

## Error in Figure

Figure 7 of the original paper [1] was mistakenly used instead of the actual and correctly measured spectrum data, illustrated below. The measured received power of the PLL was −32.64 dBm at 160 GHz with a 1 kHz view bandwidth, contrary to the reported measurement from the original article, which was −19.53 dBm at 157.82 GHz.

**Figure 7:**
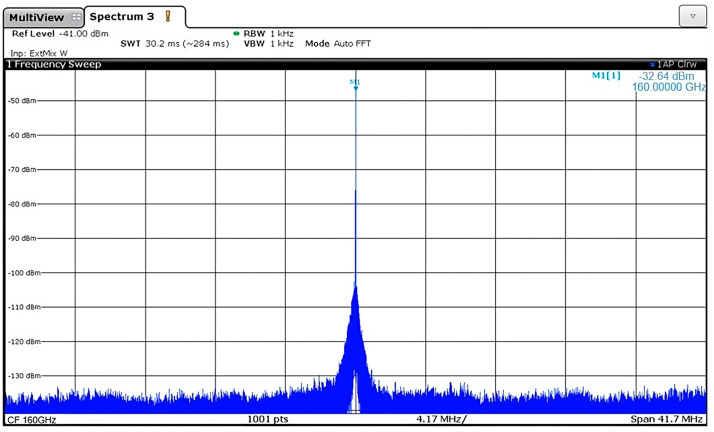
PLL output spectrum at 160 GHz.

This correction does not invalidate the other reported measurement results or the conclusions drawn from the experimental results. The authors apologize for any inconvenience this change may have caused. This correction was approved by the Academic Editor. The original publication has also been updated.

## References

[1] Kebe M., Sanduleanu M. (2023). A Low-Phase-Noise 8 GHz Linear-Band Sub-Millimeter-Wave Phase-Locked Loop in 22 nm FD-SOI CMOS. Micromachines.

